# An *In Vivo* Polymicrobial Biofilm Wound Infection Model to Study Interspecies Interactions

**DOI:** 10.1371/journal.pone.0027317

**Published:** 2011-11-04

**Authors:** Trevor Dalton, Scot E. Dowd, Randall D. Wolcott, Yan Sun, Chase Watters, John A. Griswold, Kendra P. Rumbaugh

**Affiliations:** 1 Department of Surgery, Texas Tech University Health Sciences Center, Lubbock, Texas, United States of America; 2 Department of Microbiology and Immunology, Texas Tech University Health Sciences Center, Lubbock, Texas, United States of America; 3 Research and Testing Laboratory, Lubbock, Texas, United States of America; Hopital Raymond Poincare - Universite Versailles St. Quentin, France

## Abstract

Chronic wound infections are typically polymicrobial; however, most *in vivo* studies have focused on monospecies infections. This project was designed to develop an *in vivo*, polymicrobial, biofilm-related, infected wound model in order to study multispecies biofilm dynamics and in relation to wound chronicity. Multispecies biofilms consisting of both Gram negative and Gram positive strains, as well as aerobes and anaerobes, were grown *in vitro* and then transplanted onto the wounds of mice. These *in vitro*-to-*in vivo* multi-species biofilm transplants generated polymicrobial wound infections, which remained heterogeneous with four bacterial species throughout the experiment. We observed that wounded mice given multispecies biofilm infections displayed a wound healing impairment over mice infected with a single-species of bacteria. In addition, the bacteria in the polymicrobial wound infections displayed increased antimicrobial tolerance in comparison to those in single species infections. These data suggest that synergistic interactions between different bacterial species in wounds may contribute to healing delays and/or antibiotic tolerance.

## Introduction

Infections of the dermis, including burns, surgical site infections and non-healing diabetic foot ulcers affect over a million people, cause thousands of deaths and cost billions of dollars in direct medical costs in the United States annually [1], [2], [3], [4], [5], [6], [7]. In underdeveloped nations and in areas of conflict the numbers are significantly higher [8]. Individuals with diabetes are particularly vulnerable, and among the 23.6 million diabetic patients in the U.S. (7.8% of the population), approximately 15% will develop foot ulceration during the course of their disease, and of these 14–24% will eventually undergo amputation [4], [9]. Chronically-infected diabetic foot ulcers are considered the most significant wound care problem in the United States and the world, and the exact cost of care for them is likely to be measured in billions of dollars [10]. In addition to diabetics, several other groups of immunocompromised patient populations are plagued by slow-healing and non-healing wounds. These include trauma and burn victims, cancer patients and pressure ulcers in the elderly [1], [2], [3], [4], [5], [6], [7].

Advances in molecular diagnosis have provided sensitive methods for identifying microbes present in wounds. Recently, Dowd *et al*. used pyrosequencing, shotgun Sanger sequencing and denaturing gradient gel electrophoresis to survey the microbial populations in 30 human wounds [11]. The major findings of this study were that: 1. while standard culturing techniques detected 12 different bacterial genera populating the wounds, molecular methods revealed up to 106 different bacterial genera; and 2. a large majority of the microbial wound population was made up of strict and facultative anaerobes, many of which standard culturing techniques did not detect. This study and others provided evidence of the incredible microbial diversity present in chronic wounds.

Now that molecular methods have greatly improved our vision of the wound microbiome researchers have begun to investigate the complex interspecies interactions that occur within these diverse microbial populations and test how the overall make-up of the microbial population influences healing. Experiments using a diabetic mouse wound model demonstrated that the microbial population shifts considerably over time and that these shifts are intimately associated with healing and expression of host immune-related genes [12]. In this study, the authors observed a correlation between the abundance of *Staphylococcal spp*. present and the expression of cutaneous host defense genes [12]. In addition, members of our group have previously used multivariate hierarchical clustering to evaluate the co-occurrence of particular species in chronic wound infections [13]. Bacteria genera that were detected in greater than 10% of the 40 human wounds studied were placed into 8 major clusters that were termed functional equivalent pathogroups (FEP) [13]. By identifying common bacterial consortia that frequently infect wounds, we can begin to ask whether particular consortia are more frequently associated with recalcitrant infections. This may be accomplished by correlating clinical outcomes with the presence of specific consortia, or by using animal wound models to directly test the infection sequela, healing and host-response to different consortia.

Historically, most vertebrate wound models have been utilized to examine the infection sequela of only one organism at a time. This is not surprising considering the technical difficulties associated with co-culturing diverse species that have different nutritional, oxygen and temperature requirements. Multiple species may have different growth rates leading to an unbalanced consumption of nutrients or production of metabolites, and may produce factors that are bactericidal or static to the other species. However, as discussed above, diverse populations of microbes do exist together in chronic wounds, and both microbial pathogenesis and host response are likely to be dramatically different in mono versus polymicrobial infections. Thus we sought to develop an effective *in vivo* model for studying polymicrobial wound infections. Ideally we wanted to establish a murine infection that would: 1. incorporate several important human wound pathogens; 2. remain chronically-infected over a substantial period of time; 3. be composed of both Gram negatives and positives; and 4. have at least one representative obligate anaerobic species. Once established, we wanted to utilize this model to test the hypothesis that polymicrobial infections promote wound chronicity, beyond what is seen in single-species infections.

Bacterial synergy can be defined as the cooperative interaction of two or more bacterial species to produce a result not achieved by the individual bacterium acting alone [14]. In the context of infection, this synergistic result is often an increase in virulence, as polymicrobial infections have been shown to be more virulent than infections caused by single organisms in both human and animals [15], [16]. In the current study, chronicity was defined as the propensity of the wound to remain open and infected with bacteria. While it is possible that the multiple species of bacteria present in human wounds exert synergistic effects, very few studies have attempted to evaluate these potential synergistic mechanisms *in vivo* or in the context of wound infections.

## Materials and Methods

### Bacterial strains, media, and growth conditions

*Pseudomonas aeruginosa*, strain PAO1 [17], was routinely grown aerobically at 37°C overnight in Luria Bertani (LB). For mouse infections, all overnight cultures were subcultured for 3 hours at 37°C in LB broth with aeration to an OD_600_ of approximately 0.9. For mouse infections, subcultured bacteria were serially diluted in phosphate buffer saline (PBS). For *in vitro* polymicrobial biofilms, *P. aeruginosa* PAO1 (ATCC number: BAA-47), *Enterococcus faecalis* V583 (ATCC number: 700802), *Finegoldia magna* (ATCC number: 29328) and *Staphylococcus aureus* Mu50 (ATCC number: 700699) were all grown overnight at 37°C in tryptic soy broth (TSB) with aeration in atmospheric oxygen levels for aerobes and under anaerobic conditions for anaerobes. Polymicrobial biofilms were grown at 37°C with shaking (1.5721875 × g) in glass tubes containing Bolton broth with 50% plasma and 5% freeze-thaw laked horse red blood cells, as previously described [18]. Briefly, 10 µL of each culture, that was normalized to 1×10^6^ colony forming units (CFU)/mL, was inoculated into the glass tubes. The pipette tip containing the bacterial solution was ejected into the media and acts as the surface upon which the biofilm grows. Biofilms were typically grown for 2 days under these conditions before inoculation on mouse wounds.

### Chronically-wounded mouse model

As previously described [19], [20], the chronically-wounded mouse model was used to examine *P. aeruginosa* and polymicrobial infections. Mice were anesthetized using 0.02 mL per gram weight of Nembutal stock (5 mg/mL) and shaved to expose their back. Nair® was applied to the backs of the mice for 5 minutes to remove any remaining hair. As a preemptive analgesic, 0.05 mL of lidocaine (500 µL of bupivacaine [0.25%] with 500 µL of lidocaine [2%]) was injected subcutaneously in the area to be wounded. A 1.5×1.5 cm patch of skin was then excised in a circular pattern creating a full thickness wound. The wounds were covered with a transparent, semipermeable polyurethane dressing (OPSITE, Smith and Nephew) which allowed for daily inspection of the wound, wound size determination, topical application of bacteria onto the wound, and protection from other contaminating bacteria. For monospecies infections, 10^4^ CFU of *P. aeruginosa* was injected under the OPSITE dressing and onto the wound. For polymicrobial infections, pre-formed biofilms were washed twice with sterile phosphate buffered saline (PBS), cut into equal pieces, weighed and transplanted onto the top of the wound before OPSITE application. This study was carried out in strict accordance with the recommendations in the Guide for the Care and Use of Laboratory Animals of the National Institutes of Health. The protocol was approved by the Institutional Animal Care and Use Committee of Texas Tech University Health Sciences Center (Protocol Number: 07044).

### Realtime PCR analysis to determine population distribution and bacterial load

Realtime PCR analysis to determine population distribution and bacterial load was performed as previously described [18]. Briefly, species-specific primers were designed for all four bacteria (Table 1), and used with SYBR green and 20 ng/µL total tissue DNA using a Roche 480 Real Time PCR System (Roche, Indianapolis, IN) with the following steps: 95°C for 10 minutes, and 40 cycles of 95°C for 15 seconds and 60°C for 60 seconds.

**Table 1:** Oligonucleotide sequences corresponding to 16S targets.

*P. aeruginosa*	qPCR: F, 5′-TAA GGA CAG CCA GGA CTA CGA GAA-3′; R, 5′-TGG TAG ATG GAC GGT TCC CAG AAA-3′
	FISH: (Cy3)5′-GCT GGC CTA GCC TTC-3′
*S. aureus*	qPCR: F, 5′-ATT TGG TCC CAG TGG TGT GGG TAT-3′; R, 5′-GCT GTG ACA ATT GCC GTT TGT CGT-3′
	FISH: (Cy5) 5′-GAT TCG TCT AAT GTC GTC CTT TG-3′
*F. magna*	qPCR: F, 5′- TAC TAA TGA GAG TGG CGA ACG GGT -3′; R, 5′- ATT AAT CCC GGT TTC CCG AGG CTA -3′
*E. faecalis*	qPCR: F, 5′-ACC AAG CGG CGT CAA GTA TCA AGA-3′; R, 5′-GTG TGC GCA ATC GCT CCA ATT TCT-3′
Universal 16S	qPCR: F, 5′-CCA TGA AGT CGG AAT CGC TAG-3′; R, 5′-GCT TGA CGG GCG GTG T-3′

### Bacterial visualization and imaging

Tissue sections were harvested from the wound bed and placed in formalin. Formalin-fixed tissue samples were sent to the Department of Pathology at TTUHSC for processing and hematoxylin and eosin (H&E) staining to visualize bacteria. For fluorescent *in situ* hybridization (FISH), sections were deparaffinized, microwaved in 1×0.01 M NaCitrate buffer, pH 6.0, for 15 minutes, and treated with proteinase K (20 µg/ml) at room temperature for 15 minutes. Lyophilized Cy3 and Cy5 labeled oligonucleotides (Table 1, Integrated DNA Technologies®) were solubilized in sterile water to a concentration of 1 µg/µL and stored at −20°C. For each de-parafinized section, 1.25 µL of probe in 10 µL of hybridization buffer was overlaid on top of the tissue, covered with a glass cover slip and incubated in a humid chamber at 45°C for 2 hours. Hybridization buffer was composed of: 270 µL of 5 M NaCl, 30 µL of 1 M Tris/HCl, 1.5 µL of 10% SDS, 450 µL of formamide and 750 µL of dd-H_2_O. After hybridization, unbound probe was rinsed off with washing buffer pre-warmed to 45°C: 1020 µL of 5 M NaCl, 1000 µL of 1 M Tris/HCl, 50 µL of 10% SDS, and 47.93 mL of dd-H_2_O. Samples were then submerged in washing buffer for 20 minutes, and then rinsed with deionized water. Dried samples were overlaid with mounting solution (0.5 µl of 4′,6-diamidino-2-phenylindole (DAPI 1 mg/mL) in 500 µL ProLong® Gold antifade reagent (Invitrogen)) and glass cover slips for microscopy.

Slides were analyzed using a Nikon Eclipse 80i microscope equipped with a Nikon Intensilight C-HGFI for fluorescence. Images were captured utilizing the Nikon Digital Sight DS-Fi1 and imaged with NIS Elements 3.0. When capturing the images it was necessary to adjust settings such as exposure or gain in order to reduce background or enhance clarity. Final images contained overlays of multiple image captures.

### Measuring wound closure

At the specified time point each wound was numbered and photographed adjacent to a ruler to ensure the results were not affected by the magnification of different pictures. The images were then analyzed in Adobe Photoshop to determine the wound area. The percent wound closure was determined using the following equation: (A_0_-A_t_)/A_0_×100, where A_0_ is the wound area on day 0 of the surgery and A_t_ is the area of the wound on the day of observation.

### Determining antimicrobial tolerance

Mice were anesthetized and administered chronic wounds as described above, and infected with either planktonic *P. aeruginosa* or a section of *in vitro* polymicrobial biofilm. A sterile gauze bandage was placed on top of the wound, and then covered with an OPSITE bandage. Wounds were monitored for four days and then the mice were euthanized. The antimicrobial tolerance of the bacteria adhered to the bandages was assessed as previously described [19]. Briefly, the bandages were removed, cut into 3 equal sections and weighed. One section was submerged in 100% bleach for 20 minutes, one into a 200 µg/mL solution of gentamicin for 5 hours, and one into sterile PBS for 5 hours. Bleach and gentamicin treatments were neutralized by submerging the samples in sodium ascorbate and Dey-Engley broth respectively for 10 minutes. Bandages were then transferred to sterile glass homogenization tubes containing 1 mL PBS. Samples were thoroughly homogenized and vortexed, and DNA was extracted from the resulting homogenate for realtime PCR analysis.

## Results

### In vitro-to-in vivo multispecies biofilm transplant results in an effective polymicrobial wound infection in mice

We choose four aerobic and anaerobic bacterial species most commonly detected in human wounds (*Staphylococcus aureus, Pseudomonas aeruginosa, Enterococcus faecalis and Finegoldia magna*) [11] as our representative polymicrobial cohort. During preliminary experiments, for which the data are not shown here, these strains were grown planktonically, mixed in equal parts, and then applied to murine surgical excision wounds. However, we repeatedly observed that within two days post-op, *P. aeruginosa* took over the infection (data not shown). In fact, even when *P. aeruginosa* only comprised only 1% of the starting inoculum, it still grew to 100% of the population within two days (data not shown). We were also unable to generate an infection with planktonic *F. magna*. Even when wounded mice were infected with >10^9^ CFU, we were unable to detect the obligate anaerobe by real-time PCR with species specific primers after two days (data not shown).

**Figure 1:**
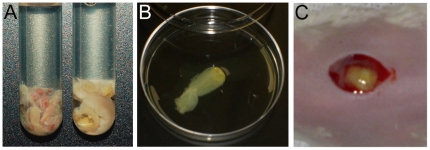
*In vitro*-to-*in vivo* polymicrobial biofilm transplant. A. Biofilms were grown *in vitro* with four different bacterial species, as described in the text. A comparison between an *in vitro* grown biofilm (left) and an actual wound debridement from a wound patient (right) are shown to demonstrate the textural similarity between this specialized media and an actual wound. B. Mature biofilms were rinsed in sterile saline and sectioned. C. biofilm sections were seeded onto the wounds of mice.

Although growing multiple species of bacteria together can be technically challenging, several model systems have been developed to generate polymicrobial biofilms *in vitro* [21], [22], [23], [24], [25]. Historically, most polymicrobial models of biofilm-related disease have focused on examining the interactions of dental microbes [24], [25], [26], [27], [28]. However, recently techniques have been developed to model the interspecies growth of microbes that make up wound biofilms as well. For example, we have previously developed a simple and effective method to grow polymicrobial biofilms *in vitro* [18], [29]. Briefly, planktonic cultures of several different bacterial species were mixed and inoculated into a glass tube containing a novel media formulation and a sterile polystyrene support for biofilm attachment (Fig. 1A) and incubated aerobically at 37°C for two to four days. This model reliably supports the growth of polymicrobial biofilms, which accurately reflect the composition of human wound infections [18], [29].

While *in vitro* models are invaluable tools for understanding the complexities of interspecies interactions, understanding how polymicrobial biofilms affect the host immune system and/or impair the healing process are crucial to the eventual development of new therapeutics. Therefore, we conducted experiments to determine if these *in vitro* grown biofilms could be used to create polymicrobial biofilm infections in mouse wounds. Biofilms consisting of *S. aureus, P. aeruginosa, E. faecalis and F. magna* were grown *in vitro* for four days, and then were aseptically removed from the tubes and rinsed in sterile saline (Fig. 1B). Biofilms were cut and a 17–23 mg section was transplanted onto the surgical excision wounds of 16 mice (Fig. 1C).

**Figure 2:**
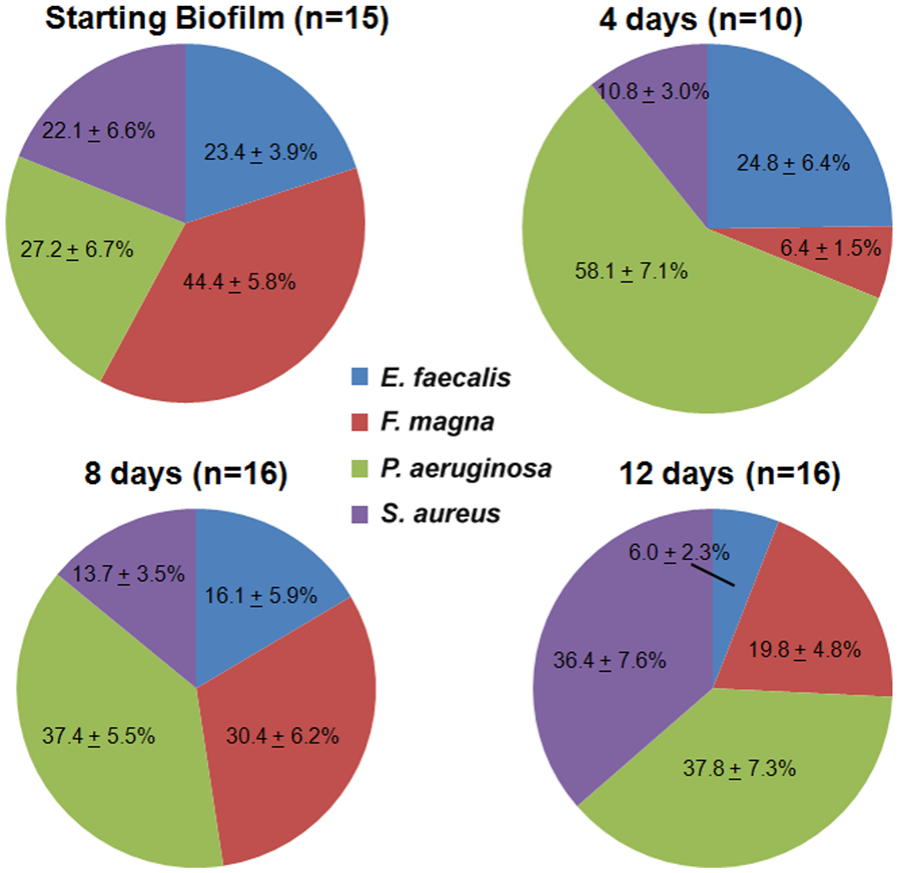
Relative population distribution of starting *in vitro*-grown biofilms and 4, 8 and 12-day wound infections. Realtime PCR was performed using species-specific primers with comparable amplification efficiencies in order to determine the relative ratio of the four different species. All mice that were infected with polymicrobial biofilms had detectable levels of all four organisms in their wounds. Average of groups ± SEM are shown.

To determine if the biofilm transplant resulted in productive polymicrobial infections we examined the population distribution within the wounds of each mouse. Realtime PCR with species specific primers (Table 1) was used in order to determine the prevalence and distribution of the four different species of bacteria in the wound tissue, as previously described [11]. Realtime PCR analysis was also performed on sections of the *in vitro* biofilms in order to determine the starting ratios of the four bacterial species. The results of this PCR analysis revealed that the population distribution of the species in the starting *in vitro* biofilms did not differ substantially from the population distribution at any of the time points examined (Fig. 2). Therefore, these data indicated that by transplanting pre-formed multi-species biofilms, we were able to create wound infections in mice that remained polymicrobial for at least 12 days.

### Monitoring in vivo polymicrobial biofilm dynamics

**Figure 3:**
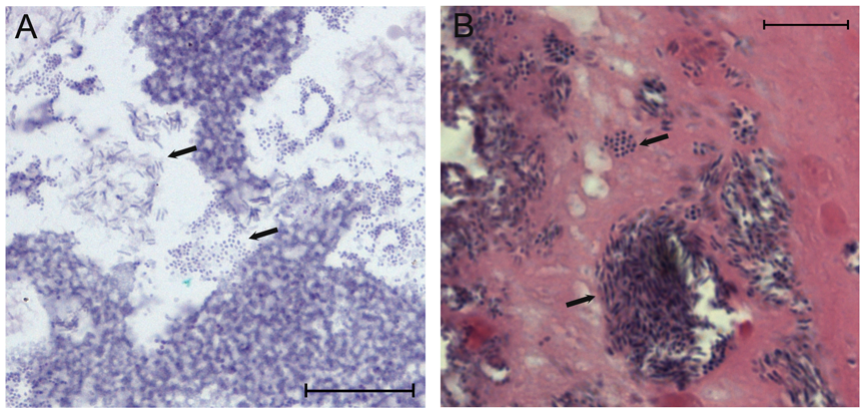
Sections of *in vitro*-grown multispecies biofilms (A) or tissue from 12-day old infected murine wounds (B) were fixed in formalin, embedded in paraffin, thin-sectioned and stained with H&E. Arrows indicate groups of morphologically distinct bacteria, scale = 10 µm.

**Figure 4:**
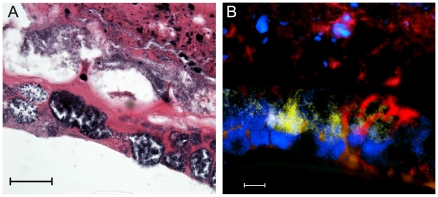
Homogeneous ‘pockets’ of bacteria were visualized along the wound margin of 12-day-old infected wounds. Wound tissue was fixed in formalin, embedded in paraffin, thin-sectioned and either stained with H&E (A) or hybridized to species-specific FISH probes (B), where *P. aeruginosa* is shown in red, *S. aureus* in yellow, and *E. faecalis*, *F. magna*, and host cell DNA are stained with DAPI (blue), scale = 10 µm.

**Figure 5:**
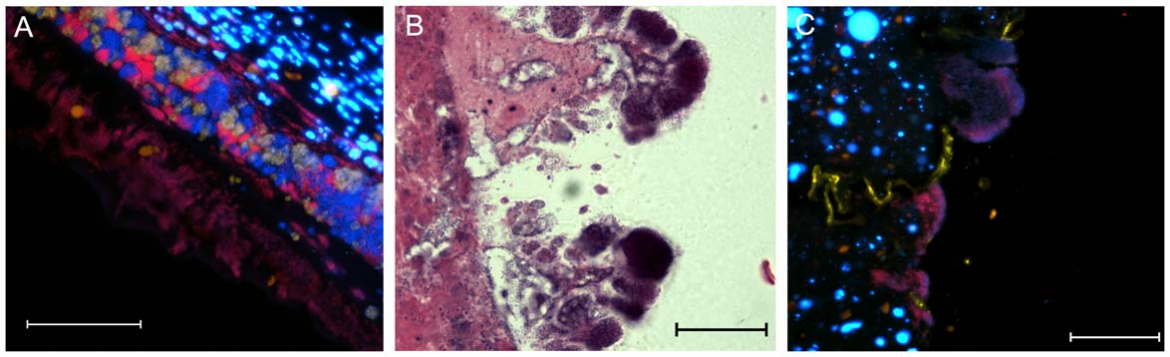
*P. aeruginosa* makes up the leading edge of the infection. (A) *In vivo* biofilms were imaged with FISH. Sections from the wound margins of 12-day-old infected mouse wounds were fixed in formalin, embedded in paraffin, thin-sectioned and mounted on slides. Sections were hybridized to a ‘target’ DNA probe complementary to a specific 16S region of the bacterial ribosomal subunit of either *P. aeruginosa* (red) or *S. aureus* (yellow) and stained with DAPI (*E. faecalis*, *F. magna*, and host cell DNA), scale = 50 µm. DAPI-stained host cell nuclei in the uninfected dermis are visible in the top right, followed by a polymicrobial-infected layer of the wound eschar, which is bordered by a layer of predominately *P. aeruginosa* extending into the wound bed. ‘Budding’ projections were visualized in the wound sections from 12-day-old polymicrobial infected mice by H&E (B) and FISH (C), scale = 10 µm. These projections extended from the leading edge of the wound margin, into the wound bed and hybridized to the *P. aeruginosa* 16S FISH probe (see in red, C).

While realtime PCR verified the relative abundance of our four representative bacterial species in mouse wounds, we wanted to investigate the dynamics of the interspecies interactions in our polymicrobial infections. Therefore we used microscopic analysis to examine the spatial distribution of the four different species in relation to each other. Microscopy of H&E-stained sections of *in vitro* biofilms and wound tissue from infected mice revealed morphologically distinct bacteria residing in close proximity to each other (Fig. 3). To distinguish between the different species of bacteria within the *in vivo* biofilms, we used fluorescent *in situ* hybridization (FISH) probes. While the different bacterial species remained in close association at all time points examined, homogeneous ‘pockets of bacteria’ were also seen with both H&E staining and FISH (Fig. 4). In tissue sections from polymicrobial biofilm-infected wounds we observed that, while *P. aeruginosa* could be seen throughout the wound bed, it was also typically seen at the leading edge of the infection, along the wound margin (Fig. 5A). The other species were typically interspersed within the tissue behind *P. aeruginosa* (Fig. 5A). Interestingly, we also observed bacteria configured in bud-like projections that lined the perimeter of the wound margin extending into the wound bed (Fig. 5A). These projections hybridized to the *P. aeruginosa* 16S FISH probe (Fig. 5B).

### Examining the ability of polymicrobial infections to promote wound chronicity

Having established a reliable method for generating polymicrobial wound infections, our next goal was to investigate whether infecting wounds with a polymicrobial population resulted in a more chronic infection than when infecting with a single species. We gave two groups of mice (16 mice/group) surgical excision wounds and infected them with either a section of polymicrobial biofilm (approx. 10^9–10^ bacteria total) or 10^4–5^ planktonic *P. aeruginosa*. Wound closure and bacterial load was assessed at 4, 8 and 12 days post-infection.

Realtime PCR with universal 16S primers was used to estimate the number of bacteria/g tissue. However, we needed to determine if we were detecting only viable bacteria or nonviable as well. Thus, we performed a set of experiments where an infecting dose of *P. aeruginosa* (10^4^ CFU) was spiked with either heat killed *E. faecalis* (10^7^ CFU) or DNA from lysed *E. faecalis* (equivalent to 10^7^ CFU). Mouse wounds were inoculated with these mixtures and wound tissue was harvested at 0, 24, 48 or 72 hours post-infection. The tissue sections were analyzed by realtime PCR with *P. aeruginosa* and *E. faecalis* 16S probes. After 24 hours neither the non-viable bacteria nor the lysed DNA were detected (data not shown), suggesting that our quantitative PCR assay was specific for viable bacteria.

**Figure 6:**
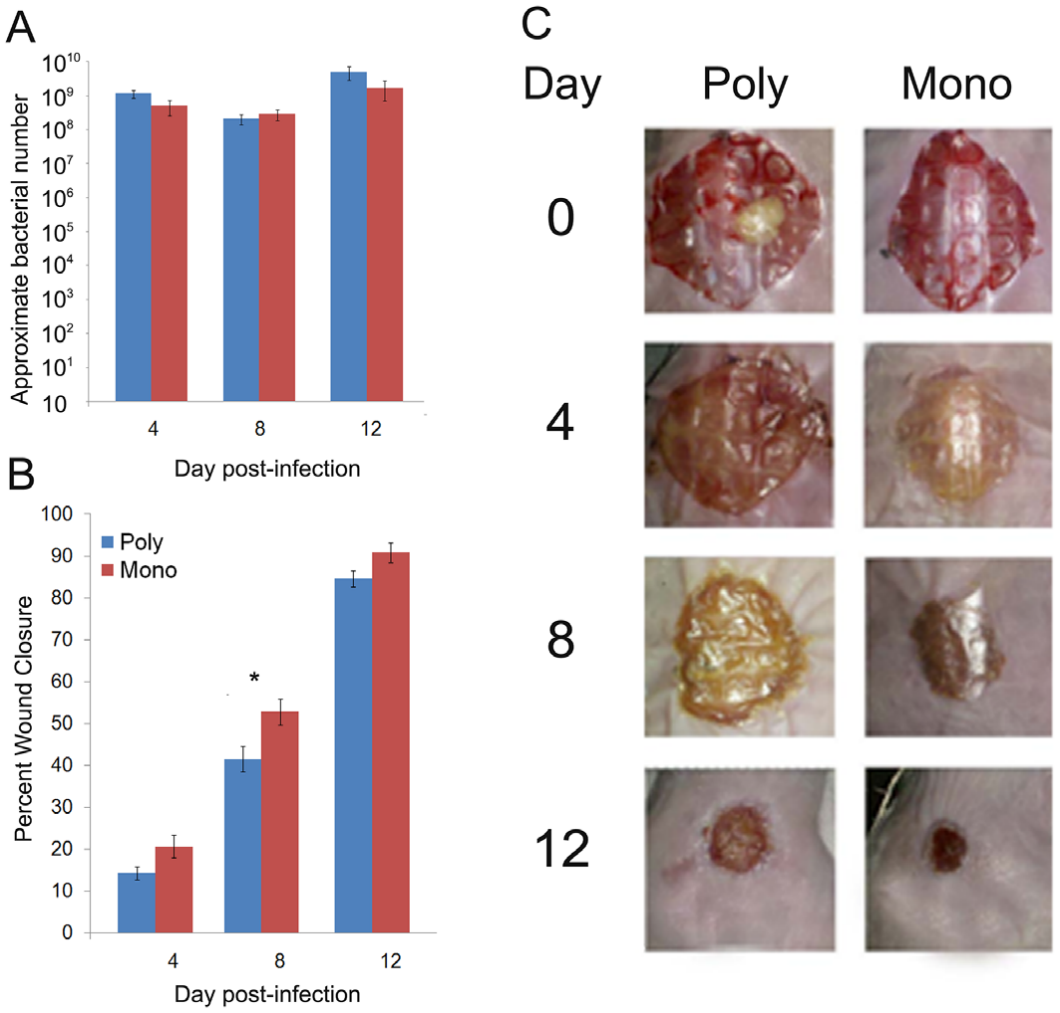
The percent closure and bacterial load was determined for wounds infected polymicrobial biofilms or *P. aeruginosa* alone. Realtime PCR analysis was used to approximate the bacterial number (A) in the infected tissue at 4, 8 and 12 days post-infection, n = 16 mice/time point. Percent wound closure (B) was determined at 4, 8 and 12 days post-infection and ANOVA with Tukey-Kramer Comparison's Test was used to determine statistical differences between groups, n = 16 mice/time point. There was no statistical difference in the bacterial load data. For the wound closure data, *p<0.05. Representative wound images are shown (C).

Although there was a considerable difference between the infecting doses used to initiate the mono and polymicrobial infections, after only 4 days we saw that the average number of bacteria in the monospecies infections, as estimated by qPCR at 4 days was 5.02×10^8^ ± 2.42×10^8^ CFU/g wound tissue, and 1.16×10^9^ ± 3.08×10^8^ CFU/g wound tissue in the polymicrobial infections (Fig. 6A). Bacterial loads in the mono and polymicrobial infections remained relatively constant (10^8–9^ CFU/g tissue) over the course of the experiment and did not differ significantly at any time point examined. In order to test our hypothesis that polymicrobial infections increase wound chronicity, we compared the wound closure of wounded mice with mono and polymicrobial infections. At all time points examined closure of the wounds with polymicrobial infections lagged behind those infected with only *P. aeruginosa* (Fig. 6B–C). However, these differences were only statistically significant at the 8-day time point (p<0.05). Taken together, these data indicate that the mere presence of multiple species in a wound do not necessarily delay wound closure.

### Measuring the antimicrobial tolerance of mono versus polymicrobial infections in situ

**Figure 7:**
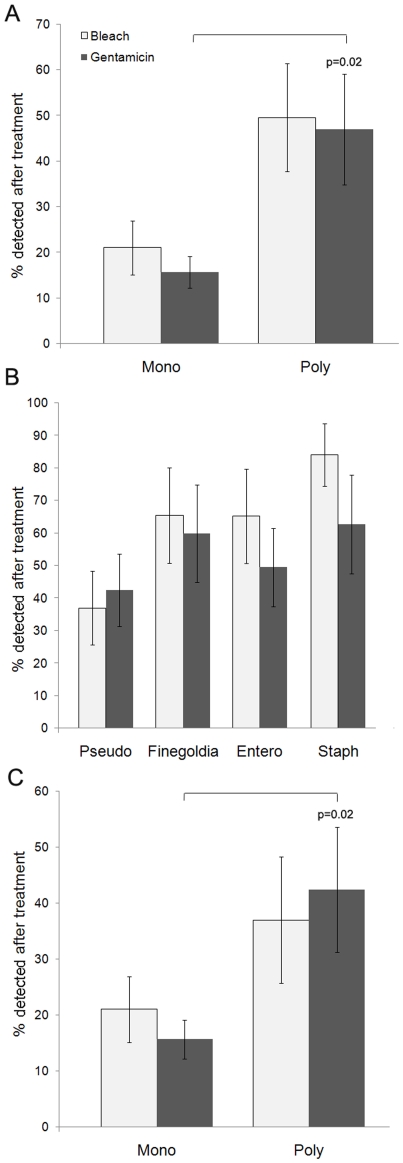
A higher percentage of bacterial cells from polymicrobial wound infections were detected after treatment with antimicrobials than those from monospecies (*P. aeruginosa*) infections (A). Percentage of each bacterial species from polymicrobial wound infections that were detected after treatment with antimicrobials (B). Percentage of *P. aeruginosa* cells, either from monospecies or polymicrobial wound infections, which were detected after treatment with antimicrobials (C). The number of bacteria in treated and untreated samples was analyzed using realtime PCR, n = 6–8 mice/group. The Mann-Whitney Test was used to determine statistical differences between groups and the two-tailed p value is shown.

Increases in antimicrobial tolerance may make some wound infections refractory to treatment and thus increase the chronicity of a wound. Therefore we examined whether the bacteria in polymicrobial wound infections displayed increased tolerance to antimicrobials in comparison to those in monospecies wound infections. We examined the efficacy of a biocide (bleach) and an antibiotic (gentamicin) to kill bacteria present in mono and polymicrobial wound infections as described in Materials and Methods. Higher numbers of bacteria remained viable after bleach (2.3-fold increase) and gentamicin (3-fold increase) treatment in the polymicrobial infection group as compared to the *P. aeruginosa* group (Fig. 7A). However, when we examined the relative tolerance of each species in the polymicrobial infection to bleach and gentamicin we observed that *P. aeruginosa* was the most susceptible (Fig. 7B). Therefore, it was not entirely surprising that a monospecies infection with *P. aeruginosa* would be more susceptible to these antimicrobial treatments than a polymicrobial infection with more tolerant species. Interestingly though, when we compared the numbers of just *P. aeruginosa* that were still viable after antimicrobial treatment in mono versus polymicrobial infections, we saw a 2-fold increase (Fig. 7C). This may indicate that being in a polymicrobial biofilm environment may make *P. aeruginosa* more tolerant to antimicrobials than when it is alone.

## Discussion

Animal models have been utilized to mimic many types of biofilm-associated infections, including those affecting the eyes, ears, heart, and bodily implants [30], [31], [32], [33]. There are also established models to study single-species biofilm-related infections in acute [34] and chronic [19], [20], [35] wounds. However, due to the technical challenges of co-culturing different species of bacteria, there is a dearth of reports investigating polymicrobial infections in wounds [36]. Therefore, the main goal of this study was to develop an *in vivo* model to study polymicrobial wound infections. We chose to focus on four species of bacteria that are commonly found in human wound infections: *S. aureus, P. aeruginosa, E. faecalis and F. magna*. Although these four species are frequently detected together in wound infections [11], our first attempts to establish polymicrobial infections by infecting wounded mice with a mixture of planktonic bacteria were unsuccessful. We observed that *P. aeruginosa* quickly became the dominate species, a phenomenon which has been seen by other investigators [22]. We were also unable to detect growth of the representative obligate anaerobe *F. magna*, when infections were initiated with planktonic bacteria.

We were able to circumvent these problems by transplanting pre-formed polymicrobial biofilms onto mouse wounds (Fig. 1). Realtime PCR analysis revealed that the resulting polymicrobial wound populations remained heterogeneous throughout the experiment (Fig. 2), and our spiking experiments, with either non-viable bacteria or DNA, demonstrated that our realtime PCR analysis was specific for detecting viable bacteria. Unlike our preliminary experiments with planktonic bacteria, the heterogeneity of the populations remained relatively constant, with no one species out-competing the others. We were also pleased to see that *F. magna* made up a large portion of the population throughout the experiment, demonstrating that obligate anaerobes are able to thrive in the aerobic/microaerobic wound environment.

The spatial distribution of different bacterial species in a polymicrobial infection could provide important clues as to the nature of their interspecies relationships. Visualization of the infected tissue with both H&E and FISH revealed that, while the different species remained in close association, small, monospecies pockets were also present (Figs. 4B and 5A). This phenomenon has also been observed in human wound biopsies [37]. The spatial distribution was similar at all time points, however we did note that as the wounds healed, more bacteria were visible in the upper eschar rather than deeper in the dermis. Unfortunately, the FISH probes for *E. faecalis* and *F. magna* showed very weak fluorescence compared to the *P. aeruginosa* and *S. aureus* probes. However, these species were detected by realtime PCR at all time points examined, and Gram positive cocci were visible upon imaging, with H&E and DAPI staining. Thus, while we were not able to visually distinguish between the two species, we were able to differentiate them from *P. aeruginosa* and *S. aureus* in our FISH analysis.

It is notable that in our experiments *P. aeruginosa* appeared to exist in a stable population with the other bacterial species. Not only did it not out-compete the others, but it grew in intimate association with them. This is contrary to our preliminary experiments using planktonic cells, and other reports [22]. It is interesting because *P. aeruginosa* produces several bactericidal factors including LasA protease (also called staphylolysin), which cleaves the pentaglycine cross-links in the peptidoglycan of *S. aureus* cells [38] and phenazine compounds, both of which are thought to enhance its competiveness over other microbes [39], [40]. Since the expression of these antimicrobial compounds are controlled by cell-density-dependent regulation (quorum sensing) [41], one possible explanation is that the *P. aeruginosa* in our polymicrobial infections did not reach sufficient cell density to initiate quorum sensing. However, *P. aeruginosa* quorum sensing autoinducers have been detected in experimental rat wounds [42] and patient debridement samples [43], making this explanation unlikely. Future experiments incorporating *P. aeruginosa* quorum sensing mutants or other isogenic mutants, deficient in the production of specific virulence factors, into the polymicrobial infections may help shed light on this issue.

We also noted that *P. aeruginosa* typically appeared at the leading edge of the infection, which is not surprising considering that it is the only motile species of the four. *P. aeruginosa* motility has been associated with tissue invasion and virulence in several murine infection models [44], [45], [46], [47], and the microbe is capable of at least three different modes of motility: swimming, swarming, and twitching. Swimming motility by planktonic *P. aeruginosa* is powered by the bacterium's single polar flagellum, but once adhered to a surface, *P. aeruginosa* moves primarily via type-IV pili (twitching motility) or surfactant-aided gliding (swarming) [48], [49]. Swarming is also flagellum-mediated, but requires the production of rhamnolipids and 3-(3-hydroxyalkanoyloxy) alkanoic acids (HAAs), which act as surfactants that help the bacterium glide across a semi-solid surface [50], [51]. Swarming motility is distinguished on soft-agar plates by the visualization of characteristic tendrils that are made when bacteria rapidly migrate from a starting inoculation point [51], [52]. Transcriptome analysis demonstrated that the swarmer cells at the tips of tendrils expressed higher levels of factors related to virulence and antibiotic resistance compared to the rest of the colony population, leading the authors to propose that these cells act as “scouts” who rapidly spread into uncolonized, nutrient-rich areas, while the biofilm population at the swarm center is the “permanent settlement” [52].

Interestingly, we visualized ‘bud-like’ projections along the leading edge of the infection, extending into the wound bed (fig. 5B). The bacteria making up these projections hybridized primarily to the *P. aeruginosa* FISH probe. While the surface of a wound may be more conducive to twitching motility, it is certainly a moist environment. Thus, it is tempting to speculate that the *P. aeruginosa* in these ‘buds’ may be part of a front of swarmer cells that are physiologically different from the *P. aeruginosa* present in the lagging polymicrobial layer. If true, it could mean that this is a hyper-virulent population of *P. aeruginosa,* which produces extracellular proteases and iron scavenging proteins involved in ‘priming’ the wound bed for colonization by the biofilm population.

Apart from establishing a model for polymicrobial wound infections, we also sought to investigate whether microbial diversity led to wound chronicity. It has been argued that the high microbial diversity seen in the oral cavity and gut, is a hallmark of commensal biofilms and indicative of a healthy microbiome, and that environmental shifts which lower diversity can lead to chronic infections [37]. In this regard, one might expect a single opportunistic pathogen to cause a more virulent infection than a consortium of common wound colonizers. However, it is also well established that synergistic interactions, especially involving anaerobes, result in disease states not accomplished by individual species alone. For example, it's hypothesized that aerobic bacteria lower local oxygen concentrations and the oxidation-reduction potential, allowing for the growth of anaerobes [53], and in return anaerobes may interfere with phagocytosis [54]. Anaerobes may also enhance the growth of other organisms in the environment as was documented in mouse abscesses where *Bacteroides* species enhanced the growth of other organisms, including *P. aeruginosa* and *S. aureus* [55]. Furthermore, Shinzato *et al*. demonstrated in a mouse model of pneumonia that mixed infections of *Streptococcus milleri* and anaerobes increased the mortality of mice compared to those of monomicrobial infections [56].

We observed that the wound closure in the mouse group given polymicrobial infections lagged significantly behind that of the monospecies-infected group at the 8 hr time point (Fig. 6B). We also observed a higher level of antimicrobial tolerance from the bacteria making up the polymicrobial infection (Fig. 7). This was not surprising considering that *P. aeruginosa* was less tolerant to the antimicrobials used than the other three species. However, it was interesting that the tolerance of the *P. aeruginosa* in the polymicrobial infections was significantly higher than that in single-species infections, suggesting that the presence of the other species imparts some protection from therapeutic agents.

Taken together, our data indicate that our approach was successful in generating polymicrobial wound infections in mice with bacterial species common to human infections. This is extremely valuable tool considering most *in vivo* wound infection models focus on studying on bacterial species at a time, and there is a limited amount of patient material, which is highly variable in the species present. With this approach many new experimental questions can be addressed: 1. How does the bacterial population make-up affect the host's response to infection? 2. Do population shifts result in slower or faster healing? 3. What environmental factors cause population shifts (diet, disease, antibiotic treatment, etc…)? By building polymicrobial populations *in vitro* that can be implanted *in vivo*, we may be able to unravel the specific roles of representative species in the wound consortia come closer to understanding these important and complex chronic infections.
